# Stock Net Entropy: Evidence from the Chinese Growth Enterprise Market

**DOI:** 10.3390/e20100805

**Published:** 2018-10-19

**Authors:** Qiuna Lv, Liyan Han, Yipeng Wan, Libo Yin

**Affiliations:** 1 School of Economics and Management, Beihang University, Beijing 100083, China; 2 Math Club Center, Acalanes High School, Lafayette, CA 94549, USA; 3 School of Finance, Central University of Finance and Economics, Beijing 100081, China

**Keywords:** network entropy, trading, DCC-threshold stock network, Chinese growth enterprise market

## Abstract

By introducing net entropy into a stock network, this paper focuses on investigating the impact of network entropy on market returns and trading in the Chinese Growth Enterprise Market (GEM). In this paper, indices of Wu structure entropy (WSE) and SD structure entropy (SDSE) are considered as indicators of network heterogeneity to present market diversification. A series of dynamic financial networks consisting of 1066 daily nets is constructed by applying the dynamic conditional correlation multivariate GARCH (DCC-MV-GARCH) model with a threshold adjustment. Then, we evaluate the quantitative relationships between network entropy indices and market trading-variables and their bilateral information spillover effects by applying the bivariate EGARCH model. There are two main findings in the paper. Firstly, the evidence significantly ensures that both market returns and trading volumes associate negatively with the network entropy indices, which indicates that stock heterogeneity, which is negative with the value of network entropy indices by definition, can help to improve market returns and increase market trading volumes. Secondly, results show significant information transmission between the indicators of network entropy and stock market trading variables.

## 1. Introduction

A complex network as a viable alternative is widely utilized in the financial system [1,2]. Since a definition of distance between stocks was proposed by Mantegna [3] with a function of correlation coefficients between pairs of stocks, numerous analyses for the evaluation and quantification of the stock network structure have been carried out by researchers, such as Vandewalle et al. [4], Bonanno et al. [5], Lee et al. [6], Chi et al. [7], Heiberger [8], and Birch et al. [9]. This proposal provides a powerful framework for understanding and modeling the static topology of interactions among stock markets.

However, initial research mainly focused on the topological analysis of static networks. With substantial economic evolution and diversified trading activities including distressed trading [10], financial market trading conditions are always changing, such as market returns and volatility. Correspondingly, the network structure constructed based on the stock time series is not permanent; therefore, the previous static network models are insufficient to capture the dynamic evolution of the stock market [11,12]. Štefan Lyócsa et al. [12] suggested that a dynamic conditional correlation approach was available for understanding the dynamic evolution mechanisms of a stock-market structure. Following this idea, some researchers considered the dynamic conditional correlation multivariate GARCH (DCC-MV-GARCH) model to find dynamic conditional correlations among stocks [13,14,15,16,17]. Some other researchers constructed correlation networks over a sliding window, such as Djauhari and Gan [18], and Papana et al. [19]. Although not strictly relevant to the issue of dynamics of stock networks, but still relevant to the analysis of dynamic correlations, we can also see some other methods on dependence analyses in the literature, such as the time-varying copula approach [20], bivariate EGARCH model [21], DSTCC-GARCH models [22], multivariate normal mixture models [23], detrended cross-correlation analysis (DCCA) [24,25], and detrended fluctuation analysis (DFA) [26].

Recently, some studies concentrated on the predictability of stock market dynamics utilizing network-based indicators. For example, Caraiani [27] and Gu [28] found that singular-value decomposition entropy has forecasting ability for the case of the DOW Jones index and the Shenzhen stock market index, respectively. Gu and Shao [29] introduced the notion of multiscale singular-value decomposition to study its explanatory ability for the Dow Jones Industrial Index. The studies above confirmed the predictive power of the global property of financial networks. Later, some studies found that the same holds true for local measures of stock networks. For example, Huang et al. [13] and Long et al. [15] found that financial institutions’ local topology structure could influence their systemic risk contributions. Caraiani [11] and Qiao et al. [16] investigated the relationships between market returns and network local measures, and showed that some of these measures have significant prediction power with respect to market returns. In a different view from all of the above, Heiberger [30] proposed a Naïve Bayes model that utilizes individual-level network measures of companies as a forecasting tool for economic development.

However, we find that little of the literature concerns itself about the indicator of network entropy in stock market network research, which is a measure of network heterogeneity to present stock market dispersion. Dispersion in the financial market can be of great significance for portfolio strategy [31]. If stock returns fluctuate differently, then it is likely to offer potential chances for portfolio diversification [32]. Besides, there is an absence in the stock market research about the relationships between network entropy and market trading activity. Therefore, we decide to construct dynamic stock market networks by the dynamic conditional correlation multivariate GARCH model and the threshold method [25]. Then, we use the dynamic network model as a tool to verify that network entropy has an effect on return and trading among stock markets.

In sum, this paper contributes to the growing literature concerning dynamic stock network analysis in the following ways. Firstly, the main innovative idea in this paper is to identify the impact of network entropy on stock market returns and trading. Specifically, we introduce two network entropy indices, Wu structure entropy (WSE) [33] and SD structure entropy (SDSE) [34], which considers both the “node difference” and “edge difference” as global network measures to demonstrate stock heterogeneity and evaluate to their relationship with market variables. The existing literature mostly relates network–structure properties with financial systemic risk contribution [13,15], stock market returns [11,16], or economic growth [26]. In this paper, we not only consider market returns as a dependent term, but also consider investigating the associations between network entropy and stock market trading volume and value that can reflect market demands. Secondly, we step further to find out the information transmission between dynamic stock networks and market returns and trading.

The remainder of the paper is organized as follows. We make a literature review for net entropy in Section 2. Section 3 displays three research hypotheses. Section 4 introduces the data of stocks and index trading variables. In Section 5, we construct dynamic correlation financial networks for the Chinese Growth Enterprise Market and study structure evolutions of dynamic stock networks. Section 6 reports the empirical results of regression test and information transmission analyses. Finally, we conclude the paper in Section 7.

## 2. Net Entropy of a Stock Market

Entropy as a measurement of system intrinsic randomness and structural complexity was initially applied to the field of communications channel by Shannon et al. [35]; since then, entropy has been extensively applied in a variety of problem areas, such as chemistry, social networks, computer science [36], and finance [37,38,39,40,41]. Entropy is especially widely employed in the issues of risk assessment [42,43,44] and market predictions [45,46,47]. Rashevsky [48], Trucco [49], and Mowshowitz [50] were the first researchers to define and investigate ‘Graph entropy’. ‘Graph entropy’ is an efficient tool to quantify the heterogeneity of a complex network, and it can be defined from two aspects. One is defined based on information theory, such as network ensemble Shannon entropy [51] and target entropy [52]. The other one called network structure entropy, focuses on the network structure, including node degree sequences, edge weights, clustering coefficients or others to demonstrate the heterogeneity of the network structure. These include Wu structure entropy defined with node difference [33], Shannon entropy, Renyi entropy, and Tsallis entropy defined with edge weight [53], and Shannon entropy defined with node degree, clustering coefficients, or centrality [54].

Although various entropies have been adopted to study financial markets [37,38,39,40,41], there are limited papers analyzing financial market heterogeneity with network entropy. Among the rare examples are Li et al. [53] who considered three types of network entropies to analyze the financial market. Nie and Song [55] introduced a standardized Rényi entropy to capture the structural differences of financial correlation-based networks. Caraiani [56] employed singular-value decomposition entropy to study comovement among various financial networks. Stutzer [57] utilized a relative entropy-based measure of minimum mutual information estimation in financial network analyses.

In this paper, we introduce two different entropy-based measures to the financial network to measure financial market diversification. Firstly, Tan and Wu [33] proposed a measure for network structure entropy based on the node degree, which is defined as Wu structure entropy. The formula is shown as follows:

(1) $$WSE=-\sum_{i=1}^{N}(k_{i}/\sum_{i=1}^{N}k_{i})\ln(k_{i}/\sum_{i=1}^{N}k_{i})\;$$

where *N* is the number of nodes in the network, $k_{i}$ denotes the degree of node *i*, and $k_{i}/\sum_{i=1}^{N}k_{i}$ represents the importance of node *i* in the network. However, Wu structure entropy (WSE) is defective when networks contain isolated nodes. To overcome this limitation, we assigned the relative importance of node *i* to be 0 if the node degree ($k_{i}=0$) equals to 0. A smaller value of network entropy means a more heterogeneous network. If the node degrees are all the same or the network is homogeneous, the maximum value of entropy ln(N) is achieved.

Further, Cai et al. [34] considered that, in order to describe a network structure more comprehensively, entropy as an indicator of network heterogeneity should simultaneously reflect two basic elements in the network: nodes and edges. They defined the “node difference” and “edge difference”. Firstly, node *i* with a degree of $k_{i}$ was selected as a controlled object. Then, the number of nodes of which the degree did not equal to $k_{i}$ could be interpreted as a measure for “node difference”, which is defined as follows:

(2) $$S_{i}=[1-p(k_{i})]N\;$$

where $p(k_{i})$ is the probability of a node degree being $k_{i}$.

Secondly, the degree of nodes that are linked by any edges should obey the distribution of kp(k); that is to say, the probability of nodes with a degree of *k* being selected is *k* times that of nodes with degree of 1. In that case, the isolated vertex would never be linked. Therefore, taking node *i* as a controlled object, the “edge difference” of node *i* can be defined as follows:

(3) $$D_{i}=k_{i}[1-p(k_{i})]N\;$$

Taking into account both the “node difference” and the “edge difference”, the structural importance of node *i* in the network is calculated as follows:

(4) $$I_{i}^{\prime }=\alpha S_{i}+\beta D_{i}=\alpha [1-p(k_{i})]N+\beta k_{i}[1-p(k_{i})]N,\alpha +\beta =1,0\leq \alpha ,\beta \leq 1\;$$

where α and β denote the weights of “node difference” and “edge difference”, respectively. If the node degrees are all the same or the network is homogeneous, the value of $I_{i}^{\prime }$ is assigned to be 1. We think the importance of “node difference” is the same as that of “edge difference”, so we let α=β=1/2.

Then, the relative importance of node *i* in the network can be calculated as follows:

(5) $$\begin{array}{ll} I_{i}=\frac{I_{i}^{\prime }}{\sum_{i=1}^{N}I_{j}^{\prime }} & =\frac{\alpha [1-p(k_{i})]N+\beta k_{i}[1-p(k_{i})]N}{\sum_{j=1}^{N}\alpha [1-p(k_{i})]N+\beta k_{i}[1-p(k_{i})]N}\\ & \approx \frac{\left(k_{i}+1)[1-p(k_{i})+\Delta \right]}{\sum_{j=1}^{N}\left\{(k_{i}+1)[1-p(k_{i})+\Delta ]\right\}},\;\Delta \sim O(\frac{1}{N^{2}}) \end{array}$$

Finally, optimized network entropy (SD structure entropy) is defined as follows:

(6) $$SDSE=-\sum_{i=1}^{N}I_{i}\log I_{i}\;$$

Compared with some other network entropy measures that consider a single network metric, such as Wu entropy, Shannon entropy, Renyi entropy, and Tsallis entropy, SD structure entropy (SDSE) considers both node and edge differences, which can describe network heterogeneity more comprehensively [34]. Considering that SDSE is an optimization of WSE, and that the two network structure entropy indices are both good measures of network heterogeneity and are widely employed to quantify the diversification of a complex system [58],we utilized both WSE and SDSE to measure the entropy of a stock network and to verify that SDSE can be more robust, to capture the information about the stock market.

The above two network entropy indices are both constructed, based on the definition of the degree, so that the network entropy indices can reflect the heterogeneity of the node degree in the network. Further, the node degree explains the importance of a node in a network. The larger the node degree is, the more important the node in the network is. Therefore, the network entropy indices present the heterogeneity of stock importance. Besides, network entropy has been proven to be a great indicator to quantify the evolving financial market organization [53].

## 3. Research Hypothesis

Based on evidence provided by the existing literature, it can be noticed that some local network measures, such as average degree, clustering coefficient, average path length, or network centrality, have an impact on the stock market returns [10,15]. These local network indicators focus on the network’s local property and reflect stock similarities in the market [11], so that they are inadequate to grab the information of the network global distribution and figure out the node differences. Network entropy makes up for these inefficiencies and they may provide a different perspective from these indicators.

Consequently, we come up with the question of whether stock network entropies have an influence on market trade-off. In the theoretical model, stock networks are constructed based on stock returns. Thus, we deduce that the stock network dynamics contain information about market return, and then network entropy should also contain the information of market return. Furthermore, market returns are causally related with trading activity [10,59,60,61], and network entropy may also be related with trading activity theoretically. Besides, intuitively, if stock returns fluctuate differently, investors may obtain more potential chances for portfolio diversification to prevent risk, and to purchase more shares to increase profits. In that case, the market trading volume will increase, and the stock return will also benefit. Urged by these assumptions, we put forward three formal hypotheses in this paper:

**Hypothesis** **1.** Market return and trading volume are negatively associated with network entropy.

**Hypothesis** **2.** When local network indicators and macro factors are controlled, market return and trading volume are still negatively influenced by network entropy.

**Hypothesis** **3.** There are significant information spillover effects between network entropy and market return or trading.

Based on the assumptions, we test the quantitative relationships between the network entropy indices and market trading variables by several linear models and information spillover effects as well, by applying a bivariate EGARCH model, which can be seen in Section 6.

## 4. Data

The Chinese Growth Enterprise Market (GEM), known as the second-board market, was established in 2010 when there were only 36 listed companies. In the past eight years, the market has become complete and has gradually matured, which attracted much attention from investors and researchers. Stock volatility and the turnover rate of the GEM are now higher than those of the main board and small and medium enterprise boards in China. Therefore, it is meaningful to choose GEM as the proxy to model stock market dynamics. We used the daily closing price data of stocks that are listed in the Growth Enterprise Index to construct dynamic correlation-based networks, and analyzed the market daily return comovement effect on market trading over a period of 1066 consecutive trading days from 29 January 2014 to 14 June 2018. The data were obtained from the WIND information database. We removed the stocks with incomplete data during the tested period and, finally, 80 stocks were reserved.

We selected two variables, namely, Growth Enterprise Market Index returns and Growth Enterprise Index trading volume, as measures of the level of trading activity [61]. The trading volume was non-stationary as indicated by unit root tests. Previous researches have shown the existence of deterministic time trends, both linear and nonlinear, in the volume data [62]. To control for these trends, and following Balcilar et al. [61], we utilized a detrended measure of volume. Specifically, we removed the trend by regressing trading volume series on a constant, (*t/T*) and (*t/T*)^2^, where *T* is the total sample size. For expositional simplicity, we hereafter refer to detrended trading volume as the trading volume. Table 1 displays the summary statistics of the index returns (*RET*) and (detrended) trading volume (*VOL*) over the sample time. As shown in Table 1, the averages of the index returns and trading volume were all positive, and the skewness and kurtosis measures showed that the distributions of returns and the detrended trading volume exhibited excess kurtosis or fat tails, as confirmed by Jarque–Bera statistics (JB). The AugmentedDickey–Fuller (ADF) tests for the two variables were all significantly negative, which indicates that the series were both stationary.

## 5. Dynamic Financial Network

In this section, we introduce the DCC-MV-GARCH model and threshold method to construct the daily GEM networks. Then, two resulting DCC-Threshold networks at their smallest and largest network density times were selected to study the network topological properties. Finally, dynamics of networks’ local topology indicators and two network-entropy indices of the financial networks are studied in the last subsection.

### 5.1. Dynamic Correlation Algorithm

#### 5.1.1. Network Construction

The DCC-MV-GARCH model proposed by Engle [63] is one of the most efficient research tools for the study of multivariable conditional heteroskedasticity, and this method offers an innovative perspective for the analysis of dynamic correlation evolution between diverse economic factors [12]. In this paper, the DCC-MV-GARCH model is applied to calculate dynamic conditional correlation coefficients among stocks. Firstly, the return of stock *i* is defined as follows:

(7) $$r_{i}(t)=\ln P_{i}(t)-\ln P_{i}(t-1)\;$$

where $P_{i}(t)$ is the closing price of stock *i* on day *t*.

As the DCC-MV-GARCH model is set under the assumption that the time sequence exerts no autocorrelation, we should estimate the residual without autocorrelation for each stock return series. The residual for each series is obtained from the ARMA model [64,65], which is shown as follows:

(8) $$r_{i,t}=c_{i}+\omega_{i,t}+\sum_{p=1}^{P}\Phi_{i,p}r_{i,t-p}+\sum_{j=1}^{q}\delta_{i,q}\omega_{i,t-q}\;$$

where $r_{i,t}$ denotes the return of stock *i* at time *t*; $c_{i}$ is the constant term; $\omega_{i,t}$ represents the stochastic error term at time *t*; $\Phi_{i,p}$ and $\delta_{i,q}$ are the coefficients in the ARMA model. We set the maximum of p and q to be 4.

Then, the conditional variance–covariance matrix ($H_{t}$) of the vector-normalized residuals $\omega_{t}$ is specified as follows:

(9) $$\omega_{t}|\Omega_{t-1}∼N(0,H_{t})\;$$

(10) $$H_{t}=D_{t}R_{t}D_{t}\;$$

where $\omega_{t}=(\omega_{t}^{1},\omega_{t}^{2},\;\cdots ,\;\omega_{t}^{N})$ is the residuals of *N* stocks at time *t*, $\Omega_{t-1}$ is the information set of $r_{t}$ at time *t*; $D_{t}=diag(\sqrt{h_{it}})$ is the (*N × N*) diagonal matrix of the conditional standard deviations of the residuals and is obtained from taking the square root of the conditional variances modeled by a univariate GARCH (1, 1) process: $h_{it}=\alpha_{i}e_{i,t-1}^{2}+\beta_{i}h_{i,}{}_{t-1}+\varpi_{i}$, in which $\alpha_{i}$ and $\beta_{i}$ are the estimated coefficients, and $e_{i,t}$ denotes the standardized residual of stock *i* that is employed in the following DCC estimations; $R_{t}$ is the dynamic conditional correlation matrix calculated by the following equation:

(11) $$R_{t}=\left[\rho_{t}^{ij}\right]=diag{\left(Q_{t}\right)}^{-1/2}Q_{t}\;diag{\left(Q_{t}\right)}^{-1/2}\;$$

(12) $$Q_{t}=(1-\kappa_{1}-\kappa_{2})\bar{Q}+\kappa_{1}(e_{t-1}e_{t-1}^{\prime })+\kappa_{2}Q_{t-1}\;$$

where $e_{t-1}$ is the standardized residual series obtained from the above univariate GARCH (1, 1) process; $\bar{Q}$ is the unconditional covariance matrix of the standard residuals series; $Q_{t}=(q_{ij,t})$ is a weighted average of a positive definite matrix that guarantees that $R_{t}$ is a correlation matrix; $\kappa_{1}$ and $\kappa_{2}$ are the coefficients estimated in the DCC model. Then, the correlation coefficient between stock *i* and stock *j* at time *t* is given by:

(13) $$\rho_{t}^{ij}=q_{ij,t}/\sqrt{q_{ii,t}q_{jj,t}}\;$$

We conducted the above estimations via the UCSD_Garch toolbox in MatLab 2016a. After calculating the DCCs between any two stocks’ return series, we can obtain the complete financial network $G_{T}(V,E)$. Compared with other proposed reduction techniques, such as minimal spanning trees [66] or planar graphs [67], the threshold approach loses no essential information [30]. The network constructed by MST is sparse, so entropy would be deficient to present the diversification of the stock market. Therefore, this paper constructs DCC–Threshold networks in the following studies. Suppose that the threshold is θ, −1≤θ≤1. If the correlation coefficient $\rho_{t}^{ij}$ is greater than or equal to θ, we add an undirected edge connecting vertices *i* and *j*.

#### 5.1.2. Network Indicators

There is evidence that local network measures have predictive ability with regard to the return of overall stock markets [11], so this paper also takes local measurements into consideration. The most commonly used measures in characterizing a network from a statistical point of view include average degree, clustering coefficient, average path length, and diameter. Various measurements also exist to explain the importance of a node in a network. The most typically employed measurements include degree centrality, closeness centrality, and betweenness centrality [68]. So, we also calculate the mean values of the node centralities as network centrality. Degree centrality is exactly proportional to the node degree, so we excluded this variable.

Average degree (*AD*) is defined as the average value of node degree. The degree $k_{i}$ of vertex *i* is defined as the number of edges in the graph incident on vertex *i*. The average degree is a measure of the average quantity of high correlations, representing network global connectivity:

(14) $$AD=\frac{1}{N}\sum_{i=1}^{N}\left(k_{i}\right)\;$$

Clustering coefficient as a measurement of network connectivity evaluates local group cohesiveness [69]. Given vertex *i*, clustering $C_{i}$ of node *i* is defined as the ratio of the number of links between the neighbors of *i* and the maximum number of such links. If the degree of node *i* is $k_{i}$ and these nodes have $e_{i}$ edges between them, we have:

(15) $$C_{i}=\frac{e_{i}}{k_{i}(k_{i}-1)/2}\;$$

If the degree of node *i* equals to 1 ($k_{i}$ = 1), the value of $C_{i}$ is set to be 1, and the clustering coefficient of isolated node is set to be zero.

Then, the average clustering coefficient (*C*) of a graph is simply calculated as follows. The larger the value of the average clustering coefficient is, the more aggregative the network is:

(16) $$C=\frac{1}{N}\sum_{i}\left(C_{i}\right)\;$$

Average path length (*L*) is defined as the average value of the shortest paths of over all the possible pairs of vertices in the network, which is utilized to measure the efficiency of network information transmission. In times of external shocks, stocks are more likely to be affected by highly correlated stocks. That is to say, stocks can be affected more easily by their neighboring nodes. Therefore, the shorter the average path length is, the more efficient the network will be. This is calculated as follows:

(17) $$L=\frac{1}{N(N-1)}\sum_{ij}\left(l_{ij}\right)\;$$

where $l_{ij}$ denotes the shortest paths between node *i* and node *j*.

Diameter (*D*) represents the largest distance between any two nodes: $D=\max_{ij}l_{ij}$, which is associated with the stability of the whole network. The larger the diameter is, the more unstable the network will be.

Closeness centrality (*CC*) is defined as the reciprocal of the total distances of a vertex to all others by Barrat et al. [68]. The higher its value is, the closer that vertex to the others is. The average value indicates the node importance of the entire network, and evaluates the capacity of the market to propagate contagion. A Low closeness centrality means that institutions depend more on others to receive messages in the network [13]. Network-average closeness centrality is defined as follows:

(18) $$ACC=\frac{1}{N}\sum_{i}\frac{1}{\sum_{j\neq i}l_{ij}}\;$$

Betweenness centrality (*BC*) has been introduced [68,69] to account quantitatively for the number of times that a node acts as a bridge along the shortest path between two other nodes. Thus, network-average betweenness centrality is obtained from the following formula:

(19) $$ABC=\frac{1}{N}\sum_{i}\sum_{j\neq k\neq i}\frac{\sigma_{jk}(i)}{\sigma_{jk}}\;$$

where $\sigma_{jk}$ is the total number of shortest paths from node *h* to node *j*, and $\sigma_{jk}(i)$ is the number of these shortest paths that pass through node *i*. A higher average betweenness centrality indicates the mean level of the node importance is higher on the whole, and more nodes have important influences on other institutions, as they stop or distort the information that passes through them.

### 5.2. Network of the Chinese Growth Enterprise Market

According to Chi [7], the network structures of the U.S. stock market constructed based on cross correlation of stock prices all display scale-free characteristics with different choices of thresholds. Heiberger [30] also confirmed that diverse levels of correlation have no influence on the network topological structure. With respect to the GEM networks, the minimum correlations among stocks over the sample period are shown to be no less than −0.35, which indicates that the negative correlations were not so strong. Therefore, negative correlations were not considered in this paper, and a value of 0.35 was selected as the lowest bound of strong correlations.

**Figure 1:**
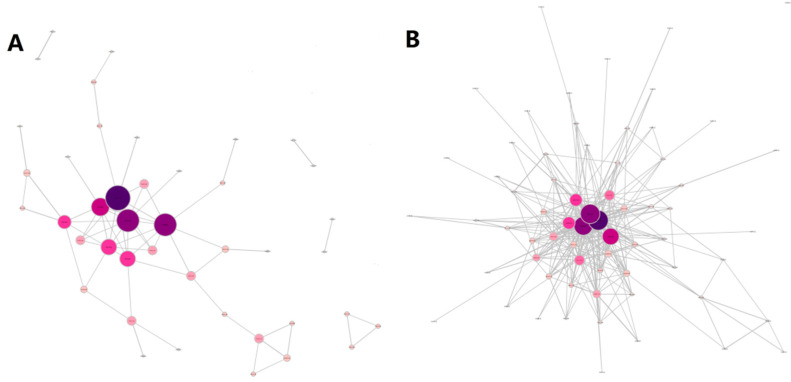
DCC-Threshold networks relative to: (**A**) the lowest network density on 20 April 2015; and (**B**) the highest network density on 8 January 2016.

Based on the data collected and the network construction method described in the previous section, we obtained 1066 consecutive 80 × 80 correlation matrixes, which corresponded to 1066 daily DCC–Threshold networks. Examples of the resulting networks estimated at two specified points in time are presented in Figure 1. The left panel shows the net structure at its smallest density time (i.e., 20 April 2015, as an example), which occurred in the prosperity period, whereas the right panel shows the graph was at its largest network density peak time (i.e., 8 January 2016), which occurred during the Chinese stock market crash from June 2015 to January 2016. The isolated vertices were removed from the network. The color and size of the node stand for its degree, which is darker and larger with the increase of its degree. The highly connected nodes were distributed in the central position, and the isolated nodes were eliminated in the network. It can be seen that the edge densities of the two networks were significantly different, while the high-degree nodes distributed in the central position were not significantly changed. The institutions with the top four node degrees on 20 April 2015 and 8 January 2016 were Nationz Technologies (300077.SZ), Risen Energy (300118.SZ), China Hualu Group (300212.SZ), and Narada (300068.SZ), which indicates that stocks with a high node degree may be stably correlated with other stocks.

In fact, a stock network is proven evidently to be scale-free in many studies. For example, Huang et al. [70] showed that China’s stock correlation network follows a scale-free network model. Kim [71] and Lee et al. [4] both found the degree distribution of Korean stock-correlation networks holds the property of a scale-free network. Chi et al. [5] and Tabak et al. [72] also came to the same findings in their analyses of U.S. and Brazil stock networks, respectively. A scale-free network is a network of which the degree distribution follows a power-law distribution, at least asymptotically. That is, P(k)~k^(−γ), where the fraction P(k) of nodes in the network has *k* connections to other nodes. In order to verify how well a power-law distribution fits the degree distribution, the fitting exponents of the power-law distribution and the *p*-value of the fitting errors are calculated for the two networks. The fitting exponent of the degree-distribution network on 20 April 2015 was shown to be 1.2644, with a significant *p*-value of 0.0005, and that of the network on 8 January 2016 was 0.6526, with a *p*-value of 6.045 × 10^−7^. The evidence indicates that the stock networks generated with the DCC-MV-GARCH model and threshold method followed a scale-free network model.

### 5.3. Indicator Discussion

**Figure 2:**
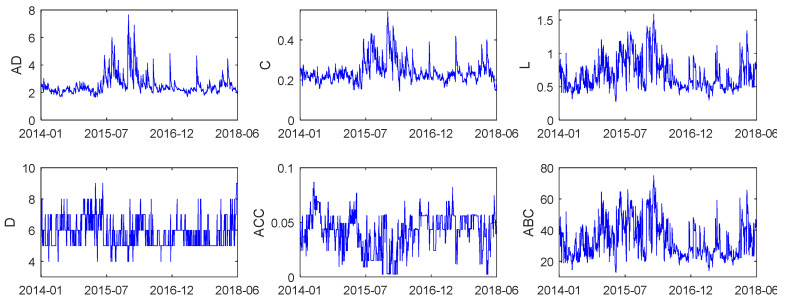
Dynamic evolutions of average degree, clustering coefficient, average path length, diameter, average closeness centrality, and average betweenness centrality in the GEM network.

Figure 2 shows the dynamic evolution of local statistical characterizations of the GEM networks. The first two figures show that the fluctuation of average-degree series behaved similarly to that of an average clustering coefficient, suggesting that network global connectivity varied consistently with local cohesiveness. Moreover, there were two major peaks in the average degree during the period from July 2015 to January 2016; when the Chinese stock market experienced obvious disaster, thousands of stocks prices plummeted, and the stock exchanges set off circuit breakers to cool off on a limit-down pause. The evidence demonstrates that GEM networks tended to have stronger connectivity during the stock market disaster, which indicates more robust interactions among stock returns during the turmoil period. This finding is exactly in line with previous research [8,11,30]. Slight peaks can also be seen in average path length and betweenness centrality during market turbulences according to the right plots in Figure 2, indicating that the scale of connected components enlarged, with more stocks joining the price comovement and acting as a bridge, and the centrality of nodes becoming much stronger than that in the prosperity period.

Furthermore, Table 2 reports the summary statistics of the network statistical measures. As shown in Table 2, the average degree ranged from 1.65 to 7.65, and the mean value of average degree was 2.5549. That is to say, when stock returns interacted most strongly, there were eight edges on each node on average, and the degree over the sample period was approximately three on average. Observing the last column, we could see that the ADF tests for each variable were all significantly negative, which indicates that the series of local network indicators were all stationary.

**Figure 3:**
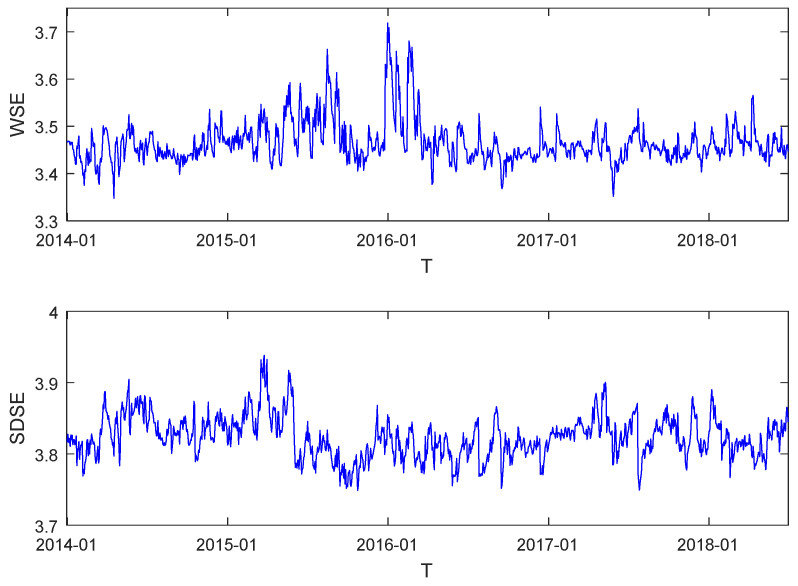
Dynamic evolution of WSE (**top** figure) and SDSE (**bottom** figure).

Figure 3 displays the dynamic fluctuations of the WSE and SDSE series of the daily DCC–Threshold networks during the sample period. The figure shows that the variations of the two network entropy indices did not fluctuate in accordance. The fluctuation trend of WSE seemed to be similar to that of the network average degree, and big peaks appeared during the period of the stock market disaster, while the bottom panel showed that big peaks appeared at the beginning of the turmoil period in SDSE, with the curve varying around a more stable mean level. The last two rows of Table 2 report the descriptive statistics of WSE and SDSE. It is shown that the mean level of SDSE was higher than that of WSE. Assuming the network is homogeneous, the entropy value is 4.3820. The maximum values of WSE and SDSE were 3.7183 and 3.9384, respectively, suggesting that daily GEM networks were always heterogeneous over the tested period. Finally, the significant results for ADF tests indicate that the two series of network entropy were both stationary.

## 6. Results

### 6.1. Network Entropy Effect on Trading and Returns

In this section, we focus on the relationship between network entropy and trading variables by estimating the following linear regression with the ordinary least-squares method to test Hypothesis 1:

(20) $$Trad_{t}=\alpha +\beta ent_{t}+\mu_{t}\;$$

where $Trad_{t}$ denotes the variables of *RET* or *VOL* mentioned in Section 2; α denotes a constant term in the intercept; $ent_{t}$ denotes indices of WSE or SDSE, and $\mu_{t}$ is the residual term.

Considering the local characteristics of networks containing significant information with regard to market dynamics [11,16,30], this paper employs network local indicators as controlled variables in the analysis of the network entropy effect on market returns and trading. Before applying linear regression models to the variables, we simply evaluated the correlations among local network indicators to avoid multicollinearity problems. The results are reported in Table 3. According to Table 3, the series of average clustering coefficient was significantly correlated with the average degree sequence, and the diameter series was significantly correlated with the average path length sequence. Correlations between WSE (SDSE) and other local network indicators were all below 0.8, which suggests that network entropy can be an additional depiction for the network’s topological structure.

Macrovariables of economic policy uncertainty (*EPU*) [73] and implied volatility index (*VIX*) [74] are also proven to contain stock-market information. Therefore, in order to ensure the robustness of the regression results, and to examine whether network entropy provides a different perspective from the local network indicators or macro factors, we introduced four groups of local network indicators, and EPU and VIX as controlled variables in the regressions to test Hypothesis 2:

(21) $$Trad_{t}=\alpha +\beta ent_{t}+\theta z_{t}+\mu_{t}\;$$

where $z_{t}=(D_{t},ACC_{t},G_{1t},G_{2t},EPU_{t},VIX_{t})$; $D_{t}$ denotes a variable of diameter; $ACC_{t}$ denotes a variable of average closeness centrality; $G_{1}$ represents a variable of average degree or average clustering coefficient, $G_{2}$ denotes a variable of average betweenness centrality or average path length; θ is a 6 × 1 coefficient vector. All estimations were conducted over the period of 29 January 2014 to 14 June 2018.

Panel A in Table 4 reports the estimation results of index return regressions with WSE as an independent variable. The evidence shows that the coefficients on WSE were all negative, and that the coefficients were significant at the 0.1 level or below in the five regressions, suggesting there was a stably significant relationship between WSE and the index returns. The index returns were also positively associated with network local measures, except for average degree, where the value was negative, suggesting its inversive impact on market returns. However, coefficients were not statistically significant. The last three rows report the F-statistics, goodness of fit ($R^{2}$), and residual standard error (RSE) of the regressions. The significant F-statistics indicate the significant regression relationship between the explanatory variables and the market returns. Comparing $R^{2}$ of the first regression with those of the last four regressions, it can be noticed that the goodness of fit improved with the addition of controlled variables. The $R^{2}$ values were approximately equal to 2%, which indicates that 2% of the overall signal of market returns were captured by the explanatory variables [74].

Panel B in Table 4 reports the estimation results, with SDSE being an independent variable, which shows that the impact of network entropy on the returns was qualitatively similar with the above estimation results in Panel A. GEM index returns were also negatively associated with SDSE, and SDSE had a significant impact on returns at the 0.1 level or below in the five regressions, which indicates that the effect of SDSE was similar with that of WSE with respect to market returns. With regard to the coefficients on local parameters, only the diameter coefficient was significant in one regression, which indicates that network local indicators had slight influence on market returns. Therefore, it can be concluded from the above analyses that network entropy provides different information from local network indicators or macro-factors, and that market returns are negatively related with the network entropy. That is, the smaller the network entropy is, the more heterogeneous the network is, and the larger the index returns will be, indicating that stock heterogeneity can benefit market returns. The three statistical indicators shown in the last three rows also exhibited similar results with those in Panel A, and the results indicate that regression accuracy improved with the addition of controlled variables.

We then turn to examine the relationship between trading volume and network entropy indices. According to Panel A of Table 5, WSE had a significantly negative effect on trading volume in the five regressions, suggesting that a decrease in WSE value would lead to an increase in trading volume. A smaller value of network entropy means a more heterogeneous network. Thus, we conclude that with the enhancement of stock market diversification, the market trading volume will increase. Panel B reports the regression results, with SDSE selected as the structure entropy variable. Similar with the regression coefficients on WSE in Panel A, estimation coefficients on SDSE were also all significantly negative in the five regressions. Therefore, we deduce that the network entropy indices had a negative effect on the index trading volume. Furthermore, evidence shows that trading volume had a significantly positive relationship with average degree, average path length, or average betweenness centrality. This finding indicates the significant effect of local indicators on the trading volume, which was worthy of detailed analysis in future research. Moreover, F-test results indicate that the explanatory variables had a significant linear relationship with the trading volume. The values of $R^{2}$ were approximately larger than 13%, which indicates that over 13% of the overall signal of trading volume could be captured by the explanatory variables, and that the estimation results of $R^{2}$ and RSE indicate that the addition of controlled variables improved the goodness-of-fit for the regressions.

To check the robustness of the results, we additionally conducted analyses with the weekly data. Table 6 reports the estimated coefficients on the network entropy indices for the weekly analyses. The evidence significantly ensures the negative relationship between network entropy indices and market returns and trading volume, which was exactly in line with the daily results. Besides, SDSE was shown to have a more robust effect on market trading and returns in weekly frequency, according to the significance of the coefficients.

In summary, the above estimation results reveal that for the Chinese Growth Enterprise Market, network entropy has a negative impact on market returns and trading volume, indicating that stock heterogeneity can help improve market returns and increase trading volume. Compared with WSE, SDSE has a more robust relationship with market trading activities in weekly frequency. Besides, it can be noticed that various network entropies are defined, to describe the degree of network heterogeneity with different metrics. Li et al. [55] found Shannon, Renyi, and Tsallis stock network entropy, evolving similarly over time. Xu [54] also discovered that network entropies constructed with node degree, clustering coefficient, and the shortest path length evolved with the same trend. Therefore, we deduce that different entropies may lead to similar results, which is worthy of detailed analysis in future research.

### 6.2. Information Transmission Analysis

Simple linear approach regression models have known limitations, so in this section, we consider the use of the optimized mean and volatility spillover-effect model to conduct a robustness test, and to further evaluate information transmission regularity between network entropy and stock-market trading to test Hypothesis 3.

If there is information spillover between network entropy indices and market-related trading variables, it can be concluded that network entropy has explanatory power on market-trading activity or that market-trading activity can influence stock network structure. Under this consideration, we used the following vector error correction model (VECM) to specify the mean spillover effect mechanism between network entropy indices and stock market trading variables. Compared with other spillover effect models, the model could estimate a bidirectional causal relationship between the variables:

(22) $$\left\{ \begin{array}{l} R_{E,t}=\mu_{E}+\sum_{i=1}^{m}\alpha_{E_{1},i}R_{E,t-i}+\sum_{i=1}^{n}\alpha_{E_{2},j}R_{I,t-i}+\alpha_{E_{3}}(\log(EN_{t-1})-\varphi_{E_{1}}\log(IN_{t-1}))+\varepsilon_{E,t}\\ R_{I,t}=\mu_{i}+\sum_{i=1}^{m}\alpha_{I_{1},i}R_{I,t-i}+\sum_{i=1}^{n}\alpha_{I_{2},j}R_{E,t-i}+\alpha_{I_{3}}(\log(IN_{t-1})-\varphi_{I_{1}}\log(EN_{t-1}))+\varepsilon_{I,t} \end{array}\right.\;$$

where $EN_{t}$, $IN_{t}$ denote the network entropy, and index trading variable on day *t*, respectively; $R_{E}=\log(EN_{t})-\log(EN_{t-1})$ and $R_{I}=\log(IN_{t})-\log(IN_{t-1})$ are the logarithmic changes of the network entropy and the index trading variable; $\alpha_{E_{1},i}$ and $\alpha_{I_{1},i}$ measure their own lagged returns effects. $\alpha_{E_{2},j}$ and $\alpha_{I_{2},j}$ measure the mean spillover effects between network entropy and index trading variable, respectively; $\alpha_{E_{3}}$ and $\alpha_{I_{3}}$ reflect the value adjustment toward the long-run equilibrium relationship between network entropy and the index trading variable.

The estimation results for the mean spillover effects between network entropy indices and index trading variables are reported in Panel A of Table 7. A number of observations are reported with t-statistics, given in the parentheses for each estimated coefficients.

Firstly, we focused on the cross coefficients of lagged series that measure the short-term influence of one variable on another. It can be seen from Table 7 that the estimators of $\alpha_{I_{2},j}$ were significantly negative for the mean spillover effects from WSE to index returns and trading volume, which indicates that WSE had a significantly negative effect on market returns and trading volume. Estimation coefficients of $\alpha_{E_{2},j}$ were negative for all mean spillover effects from market return and trading volume to WSE. The coefficient was statistically significant with respect to trading return, which indicated that there is a significant spillover effect from market returns to WSE. As for SDSE, the lagged series were almost significant in the estimation results of the own lagged return effects and mean spillover effects. The index returns and trading volume both associated negatively with SDSE. Therefore, we conclude that mean spillover effects between WSE/SDSE and trading variables were similar, and, generally, a percentage change in the network entropy indices would lead to a negative percent change in market returns and market-trading volume.

Secondly, we concentrated on the estimation results of the error-correction terms that measured adjustment to the long-run equilibrium relationships between network entropy indices and index trading variables. The results report that, for the index trading variables mean equation, all of the coefficients on the error-correlation terms were significant at the 1% level with respect to WSE and SDSE. In this case, equity market-trading activity and network entropy were informationally efficient in the long run.

Knowing that volatility is also a most significant source of information, an estimation of the volatility spillover effect would be of great help to further understanding the information transmission procedure between stock-return diversification and market-trading activity. Toward this objective, we considered using conditional variance equations for volatility transmission between the network entropy and market-trading variables by referring to the bivariate-EGARCH model [21,75]. Therefore, residuals $\varepsilon_{E,t}$ and $\varepsilon_{I,t}$ generated from the VECM model, were substituted into the conditional variance equations of the bivariate-EGARCH model, as follows:

(23) $$\begin{array}{ll} \ln\sigma_{E,t}^{2}= & v_{E}+\sum_{k=1}^{l}\beta_{E_{1},k}(|\frac{\varepsilon_{E,t-k}}{\sigma_{E,t-k}}-E(\frac{\varepsilon_{E,t-k}}{\sigma_{E,t-k}})|+\gamma_{E,k}\frac{\varepsilon_{E,t-k}}{\sigma_{E,t-k}})\\ & +\sum_{r=1}^{q}\beta_{E_{2},r}(|\frac{\varepsilon_{I,t-r}}{\sigma_{I,t-r}}-E(\frac{\varepsilon_{I,t-r}}{\sigma_{I,t-r}})|+\gamma_{I,r}\frac{\varepsilon_{I,t-r}}{\sigma_{I,t-r}})+\sum_{f=1}^{s}\beta_{E_{3},f}\ln(\sigma_{I,f}^{2}) \end{array}$$

(24) $$\begin{array}{ll} \ln\sigma_{I,t}^{2}= & v_{I}+\sum_{k=1}^{l}\beta_{I_{1},k}(|\frac{\varepsilon_{I,t-k}}{\sigma_{I,t-k}}-E(\frac{\varepsilon_{I,t-k}}{\sigma_{I,t-k}})|+\gamma_{I,k}\frac{\varepsilon_{I,t-k}}{\sigma_{I,t-k}})\\ & +\sum_{r=1}^{q}\beta_{I_{2},r}(|\frac{\varepsilon_{E,t-r}}{\sigma_{E,t-r}}-E(\frac{\varepsilon_{E,t-r}}{\sigma_{E,t-r}})|+\gamma_{E,r}\frac{\varepsilon_{E,t-r}}{\sigma_{E,t-r}})+\sum_{f=1}^{s}\beta_{I_{3},f}\ln(\sigma_{E,f}^{2}) \end{array}$$

where $\frac{\varepsilon_{I,t}}{\sigma_{I,t}}$ and $\frac{\varepsilon_{E,t}}{\sigma_{E,t}}$ are the standardized innovations. In addition, $\beta_{E_{2},r}$ and $\beta_{I_{2},r}$ measure the volatility spillover effects between the network entropy and index-trading variables, respectively. $\beta_{E_{3},f}$ and $\beta_{I_{3},f}$ measure the volatility persistence degree for the network entropy and index-trading variables. Volatility is more persistent if the value is closer to 1, which indicates that even greater volatility follows high volatility in the same direction. $\gamma_{E,k}$, $\gamma_{I,r}$, $\gamma_{I,k}$, and $\gamma_{E,r}$ measure the volatility asymmetry effect. If these coefficients are not significantly equal to zero, an asymmetry effect is present. In this paper, *k* and *r* are set to be 1.

Estimation results of the volatility spillover effects between network entropy indices and stock market trading variables are reported in Panel B of Table 7. The significant estimators of $\beta_{E_{2},j}$ and $\beta_{I_{2},j}$ indicate the rejection of the hypothesis, indicating that there were significant volatility spillover effects between WSE/SDSE and GEM index-trading variables. Besides, it can also be noticed that there were significant own lagged volatility effects between SDSE and the market trading volume, and the asymmetry effects were present between the network entropy and certain trading variables. In summary, the estimation results indicate that fluctuations in network entropy and market-trading activity have significant impacts on each other.

## 7. Conclusions

Network entropy is an indicator quantifying the heterogeneous degree of a network. The smaller the value of network entropy is, the more heterogeneous the network is. Applying this concept to stock network analysis can provide us with a new perspective on understanding stock market diversification. The main novelty in our paper is that we introduce net entropy as an indicator of network heterogeneity into a stock network, and subsequently reveal net heterogeneity with financial meanings. Specifically, we consider two indices of network entropy, WSE and SDSE, as indicators of network heterogeneity to present market diversification and then report an empirical investigation on the association between network entropy and stock market returns and trading in GEM, which was selected for containing more active stocks with higher return volatilities.

Before performing empirical estimation, we first constructed dynamic GEM networks consisting of 1066 consecutive daily nets by employing the dynamic conditional correlation multivariate GARCH (DCC-MV-GARCH) model and setting correlation thresholds. We analyzed the dynamic evolution of topological structures of daily correlation networks, and found that the network entropy indices increased in the period of the market crash, indicating that stock networks tend to be more homogeneous during times of turmoil while staying steadily small during normal periods. Weak correlations between network entropy indices and other network local indicators verify that network entropy can be a prominent description of the network structure in terms of market heterogeneity.

Based on the constructed networks, we related market trading variables with network entropy by linear regression analyses. We also explored the information-transmission mechanism between network entropy and market trading variables in bivariable EGARCH modeling. Our estimation results showed that a percent change in the value of entropy would lead to a negative percent change in the market returns and trading volumes at both daily and weekly frequencies. The explanatory ability of SDSE is more efficient than WSE in weekly frequencies, suggesting that SDSE contains more information about market-trading variables. Moreover, there are significant mean and volatility spillover effects between network entropy and market returns and trading, and local network indicators are shown to have slight predictive power for market returns.

In sum, the above quantitative analyses tell us that the smaller the value of the network entropy indicator, the higher the market returns and trading volume will be. That is, the more heterogeneous the stock network is, the higher market returns are, and the more active the market activity will be, which is exactly in line with the fact that portfolio diversification can help to benefit the stock market trading activity. Additionally, the results also offer references for market investors in portfolio diversification to prevent risk across stock markets.

## Figures and Tables

**Table 1:** Summary statistics of trading variables.

Variable	Mean	Min	Max	Std. Dev.	Skewness	Kurtosis	JB	ADF
index returns (*RET*)	0.0001	−0.0933	0.0691	0.0212	−0.6410	5.7852	411.5000 ***	−9.3918 ***
*VOL*	0.0002	−2.7224	3.7566	1.0761	0.7633	3.4216	6.5940 ***	−3.9248 **

Notes: *VOL* denotes the series of detrended trading volume (billions of CNY). Lags in ADF tests are selected by Akaike Information Criterion (AIC). ** and *** indicate significance at the 5% and 10% levels.

**Table 2:** Summary statistics of network measurements and macro factors.

Variable	Mean	Min	Max	Std. Dev	Skewness	Kurtosis	JB	ADF
AD	2.5549	1.6500	7.6500	0.7528	2.6726	12.0187	4881.8 ***	−4.6724 ***
C	0.2433	0.1447	0.5420	0.0571	1.7325	6.6846	1136.3 ***	−4.8559 ***
L	0.6818	0.2785	1.5880	0.2332	1.0950	3.7486	237.9 ***	−4.6379 ***
D	5.8565	4.0000	9.0000	0.9420	0.8081	3.2974	119.9 ***	−5.0254 ***
ACC	0.0417	0.0023	0.0869	0.0167	−0.4451	2.8129	36.7 ***	−4.9271 ***
ABC	33.6322	12.8250	75.0500	11.7622	0.8625	3.0327	132.2 ***	−4.7012 ***
WSE	3.4657	3.3476	3.7183	0.0456	1.8426	8.3604	1879.5 ***	−5.8809 ***
SDSE	3.8235	3.7489	3.9384	0.0298	0.3624	3.5176	35.2 ***	−5.7875 ***
EPU	0.0002	−1.8587	3.2156	0.5588	0.2742	1.4854	109.7 ***	−14.8010 ***
VIX	−0.0001	−0.3411	0.7682	0.0823	1.3573	10.2224	4919.4 ***	−12.4900 ***

Notes: AD denotes the average degree, C represents the average clustering coefficient, L represents the average path length, D represents the diameter, ACC represents the average closeness centrality, and ABC the represents average betweenness centrality. *** indicate significance at the 10% levels.

**Table 3:** Correlation analysis on thenetwork indicators.

	AD	C	L	D	ACC	ABC	WSE	SDSE
AD	1.000	0.939	0.769	−0.212	−0.719	0.658	0.747	−0.447
C		1.000	0.674	−0.290	−0.661	0.559	0.665	−0.497
L			1.000	0.282	−0.588	0.986	0.777	−0.179
D				1.000	−0.096	0.411	0.036	0.332
ACC					1.000	−0.821	−0.532	0.407
ABC						1.000	0.723	−0.103
WSE							1.000	0.205
SDSE								1.000

Notes: AD denotes the average degree, C represents the average clustering coefficient, L represents the average path length, D represents the diameter, CC represents the average closeness centrality, and BC represents the average betweenness centrality.

**Table 4:** Regressions of network indicators on index returns.

	Index Returns				
	Model 1	Model 2	Model 3	Model 4	Model 5
	**Panel A: Wu Structure Entropy (WSE)**				
WSE	−0.0417 * (−1.87)	−0.0543 ** (−1.85)	−0.0540 ** (−1.92)	−0.0602 *** (−2.09)	−0.0623 ** (−2.33)
D		0.0007 (0.64)	0.0004 (0.36)	0.0013 (1.24)	0.0008 (0.74)
ACC		0.1001 (1.06)	0.1001 (1.11)	0.1127 (1.19)	0.1255 (1.41)
AD		−0.0003 (−0.13)	0.0005 (0.26)		
C				0.0190 (0.90)	0.0241 (1.18)
L		0.0163 (1.54)		0.0135 (1.32)	
ABC			0.0003 * (1.68)		0.0003 * (1.65)
EPU		−0.0016 (−1.38)	−0.0016 (−1.39)	−0.0016 (−1.37)	−0.0016 (−1.38)
VIX		−0.0226 ** (−2.88)	−0.0226 ** (−2.88)	−0.0227 *** (−2.90)	−0.0227 *** (−2.90)
α	0.1457 * (1.88)	0.1698 * (1.79)	0.1698 * (1.85)	0.1830 ** (1.95)	0.1906 *** (2.17)
F	3.50 ***	2.8380 **	2.9050 ***	2.9540 ***	3.0980 ***
$R^{2}$ (%)	0.69	2.10	2.10	2.10	2.10
RSE	0.03	0.02	0.02	0.02	0.02
	**Panel B: SD Structure Entropy (SDSE)**				
SDSE	−0.0604 ** (−1.97)	−0.0645 ** (−2.24)	−0.0643 ** (−2.27)	−0.0517 * (−1.78)	−0.0558 * (−1.94)
D		0.0011 (1.06)	0.0009 (0.78)	0.0019 * (1.92)	0.0016 (1.45)
ACC		0.0960 (1.08)	0.0975 (1.14)	0.0726 (0.82)	0.0961 (1.12)
AD		−0.0029 (−1.42)	−0.0023 (−1.38)		
C				0.0049 (0.22)	−0.0070 (−0.35)
L		0.0123 (1.37)		0.0039 (0.51)	
ABC			0.0002 (1.50)		0.0001 (0.90)
EPU		−0.0016 (−1.37)	−0.0016 (−1.37)	−0.0016 (−1.36)	−0.0016 (−1.36)
VIX		−0.0230 *** (−2.94)	−0.0230 *** (−2.94)	−0.0228 *** (−2.91)	−0.0228 *** (−2.91)
α	0.2298 * (1.96)	0.2357 ** (2.18)	0.2354 ** (2.21)	0.1824 * (1.66)	0.1980 * (1.82)
F	3.87 **	3.067 ***	3.1200 ***	2.7790 ***	2.8590 ***
$R^{2}$ (%)	0.53	2.10	2.10	2.10	2.10
RSE	0.02	0.02	0.02	0.02	0.02

Notes: *, **, *** indicate significance at the 1%, 5%, and 10% levels. Numbers in the parentheses are t-statistic values.

**Table 5:** Regressions of network indicators on index trading volume.

	Trading Volume				
	Model 1	Model 2	Model 3	Model 4	Model 5
	**Panel A: Wu Structure Entropy (WSE)**				
WSE	−3.0949 ***	−5.5598 *** (−3.86)	−4.8069 ***	−5.406 ***	−4.2074 ***
	(−4.31)		(−3.46)	(−3.81)	(−3.18)
D		−0.0251	−0.0299	−0.0349	−0.0438
		(−0.48)	(−0.54)	(−0.69)	(−0.79)
ACC		6.2542	3.422	6.126	2.2922
		(1.34)	(0.77)	(1.31)	(0.52)
AD		0.0691	0.1787 **		
		(0.72)	(1.98)		
C				0.4438	1.4247
				(0.43)	(1.41)
L		2.2785 ***		2.3569 ***	
		(4.38)		(4.68)	
ABC			0.0343 ***		0.0354 ***
			(3.92)		(4.06)
EPU		−0.0184	−0.0189	−0.0185	−0.0191
		(−0.32)	(−0.33)	(−0.33)	(−0.33)
VIX		0.3686	0.3729	0.3671	0.3688
		(0.95)	(0.96)	(0.95)	(0.95)
α	10.7257 ***	17.4241 ***	15.0808 ***	16.9691 ***	13.2044 ***
	(4.313)	(3.72)	(3.32)	(3.67)	(3.04)
F	18.61 ***	13.44 ***	13.86 ***	13.39 ***	13.56***
$R^{2}$ (%)	1.72	8.17	7.84	8.14	7.67
RSE	1.07	1.04	1.04	1.04	1.04
	**Panel B: SD Structure Entropy (SDSE)**				
WSE	−5.1960 ***	−6.0595 ***	−5.6154 ***	−6.1642 ***	−5.8321 ***
	(−4.74)	(−4.27)	(−4.02)	(−4.31)	(−4.12)
D		0.0171	0.0102	0.0197	0.001
		(0.34)	(0.19)	(0.4)	(0.02)
ACC		5.02	3.04	4.6477	3.0363
		(1.15)	(0.72)	(1.07)	(0.72)
AD		−0.1842 *	−0.069		
		(−1.85)	(−0.83)		
C				−2.164 *	−1.1692
				(−1.96)	(−1.18)
L		1.8007 ***		1.6722 ***	
		(4.08)		(4.36)	
ABC			0.028 ***		0.0286 ***
			(3.7)		(4.04)
EPU		−0.0164	−0.017	−0.0166	−0.0172
		(−0.29)	(−0.3)	(−0.29)	(−0.3)
VIX		0.3326	0.3374	0.3408	0.3396
		(0.86)	(0.87)	(0.88)	(0.88)
α	19.8680 ***	22.1017 ***	20.5173 ***	22.6462 ***	21.4911 ***
	(4.74)	(4.15)	(3.91)	(4.2)	(4.01)
F	22.42 ***	13.96 ***	13.51 ***	14.02 ***	13.62 ***
$R^{2}$ (%)	2.06	8.46	8.21	8.49	8.27
RSE	1.07	1.03	1.03	1.03	1.03

Notes: *, **, *** indicate significance at the 1%, 5%, and 10% levels. Numbers in the parentheses are t-statistic values.

**Table 6:** Regressions of network indicators on market variables for weekly data.

	Model 1	Model 2	Model 3	Model 4	Model 5
	**Panel A: Index Returns**				
WSE	−0.0140 (−0.20)	−0.2935 (−1.76)	−0.2704 (−1.64)	−0.2922 * (−1.87)	−0.2426 (−1.65)
SDSE	−0.2358 * (−1.74)	−0.3097 (−1.49)	−0.2988 * (−1.95)	−0.3260 * (−1.65)	−0.3113 (−1.50)
	**Panel B: Trading Volume**				
WSE	−5.0350 (−1.33)	−18.9196 * (−1.97)	−17.2429 * (−1.83)	−18.2344 * (−1.94)	−15.1681 (−1.62)
SDSE	−12.989 ** (−2.33)	−18.0666 ** (−2.03)	−17.1747 ** (−2.15)	−18.2988 ** (−2.04)	−17.5346 ** (−2.18)

Notes: * and ** indicate significance at the 1% and 5% levels. Numbers in the parentheses are t-statistic values.

**Table 7:** Estimated results of spillover effects between the network entropy and the GEM index variable.

	WSE/RET	WSE/VOL	SDSE/RET	SDSE/VOL
**Panel A: Conditional Mean Equations** Own lagged returns effects				
$\alpha_{E_{1},1}$	0.0154 (1.33)	−0.1239 *** (−4.39)	0.0115 (0.91)	−0.1117 *** (−4.06)
$\alpha_{I_{1},1}$	0.0191 * (1.95)	−0.0624 (−0.06)	0.0287 * (1.65)	−1.3401 ** (−2.69)
Mean spillover effects				
$\alpha_{E_{2},1}$	−0.1396 *** (−4.81)	−0.0005 (−0.72)	−0.1246 *** (−4.47)	−0.0079 *** (−6.26)
$\alpha_{I_{2},1}$	−0.0826 (−1.07)	−0.2100 *** (−7.72)	−0.1208 (−0.94)	−0.2628 *** (−10.89)
Error-correction terms				
$\alpha_{E_{3}}$	0.0002 (0.56)	0.0001 (0.57)	0.0001 (0.44)	0.0001 (0.19)
$\alpha_{I_{3}}$	−0.0036 * (−1.98)	−0.0452 *** (−4.55)	−0.0039 * (−1.72)	−0.0451 *** (−4.51)
**Panel B: Conditional Variance Equations** Own lagged volatility effects				
$\beta_{E_{1},1}$	0.0034 (1.14)	−0.0003 (−0.63)	0.0031 (1.37)	0.1903 ** (2.75)
$\beta_{I_{1},1}$	0.0107 *** (3.26)	0.0002 (0.31)	0.0048 (1.26)	0.1986 *** (3.09)
Volatility spillover effects				
$\beta_{E_{2},1}$	0.4817 *** (8.57)	0.2182 *** (2.81)	0.0914 ** (2.13)	0.4556 *** (8.12)
$\beta_{I_{2},1}$	0.2513 *** (5.84)	0.2521 *** (4.11)	0.1665 *** (2.96)	0.1428 * (1.94)
Asymmetry for volatility				
$\gamma_{E,1}$	−9.5389 *** (−70.61)	−150.4575 ** (−2.05)	13.6072 *** (9.76)	−0.2718 (−1.27)
$\gamma_{I,1}$	−0.1886 *** (−3.45)	0.1574 (0.90)	−0.2426 *** (−3.73)	0.1362 (1.32)

Notes: This table reports estimation results for parameters in Equations (22)–(24). *, **, *** indicate significance at the 1%, 5%, and 10% levels.
